# Screening Antibacterial Photodynamic Effect of *Monascus* Red Yeast Rice (Hong-Qu) and Mycelium Extracts

**DOI:** 10.1007/s00284-024-03725-6

**Published:** 2024-05-21

**Authors:** Marketa Husakova, Viviana Teresa Orlandi, Fabrizio Bolognese, Barbora Branska, Petra Patakova

**Affiliations:** 1 Department of Biotechnology, University of Chemistry and Technology Prague, Technicka 5, 160 00 Prague, Czech Republic; 2 Department of Biotechnologies and Life Sciences, University of Insubria, Via JH Dunant 3, 21100 Varese, Italy

## Abstract

The fungus *Monascus* is a well-known source of secondary metabolites with interesting pharmaceutical and nutraceutical applications. In particular, *Monascus* pigments possess a wide range of biological activities (e.g. antimicrobial, antioxidant, anti-inflammatory or antitumoral). To broaden the scope of their possible application, this study focused on testing *Monascus* pigment extracts as potential photosensitizing agents efficient in antimicrobial photodynamic therapy (aPDT) against bacteria. For this purpose, eight different extracts of secondary metabolites from the liquid- and solid-state fermentation of *Monascus purpureus* DBM 4360 and *Monascus* sp. DBM 4361 were tested against Gram-positive and Gram-negative model bacteria, *Bacillus subtilis* and *Escherichia coli* and further screened for ESKAPE pathogens, *Staphylococcus aureus* and *Pseudomonas aeruginosa*. To the bacterial culture, increasing concentration of extracts was added and it was found that all extracts showed varying antimicrobial activity against Gram-positive bacteria in dark, which was further increased after irradiation. Gram-negative bacteria were tolerant to the extracts’ exposure in the dark but sensitivity to almost all extracts that occurred after irradiation. The *Monascus* sp. DBM 4361 extracts seemed to be the best potential candidate for aPDT against Gram-positive bacteria, being efficient at low doses, i.e. the lowest total concentration of *Monascus* pigments exhibiting aPDT effect was 3.92 ± 1.36 mg/L for *E.* *coli*. Our results indicate that *Monascus* spp., forming monascuspiloin as the major yellow pigment and not-forming mycotoxin citrinin, is a promising source of antimicrobials and photoantimicrobials.

**Supplementary Information:**

The online version contains supplementary material available at 10.1007/s00284-024-03725-6.

## Introduction

The *Monascus* fungi are well known mainly in Asian countries for centuries. The most significant *Monascus* product is the red yeast rice (RYR) alias hong-qu (the Chinese term for RYR), i.e. rice fermented by *Monascus purpureus* or different *Monascus* species. RYR can be used, depending on the used *Monascus* species or strain and the fermentation method, for food colouring, as a food supplement or as an inoculum for another fermentation [1]. Lipid lowering food supplements containing RYR with monacolin K and its derivatives (monacolin K being synonym to lovastatin) are famous throughout the world [2]. However, the production of monacolin K is not inherent in all strains of *Monascus* and is achieved under specific cultivation conditions. In addition to solid surface cultivation (SSC), also submerse liquid cultivation (SLC) can be used for *Monascus* culture [3]. The main secondary metabolites are *Monascus* pigments (MPs) which are traditionally used as food colourants for meat and fish processed products or candies. It is not possible to conceal that there is a potential risk associated with all *Monascus* products, which is the ability of some *Monascus* strains to produce mycotoxin citrinin. Citrinin production can be eliminated or reduced significantly by different strategies [4], yet citrinin concentration in sensitive foods and food supplements must be always checked by a convenient analytical method [5].

**Fig. 1:**
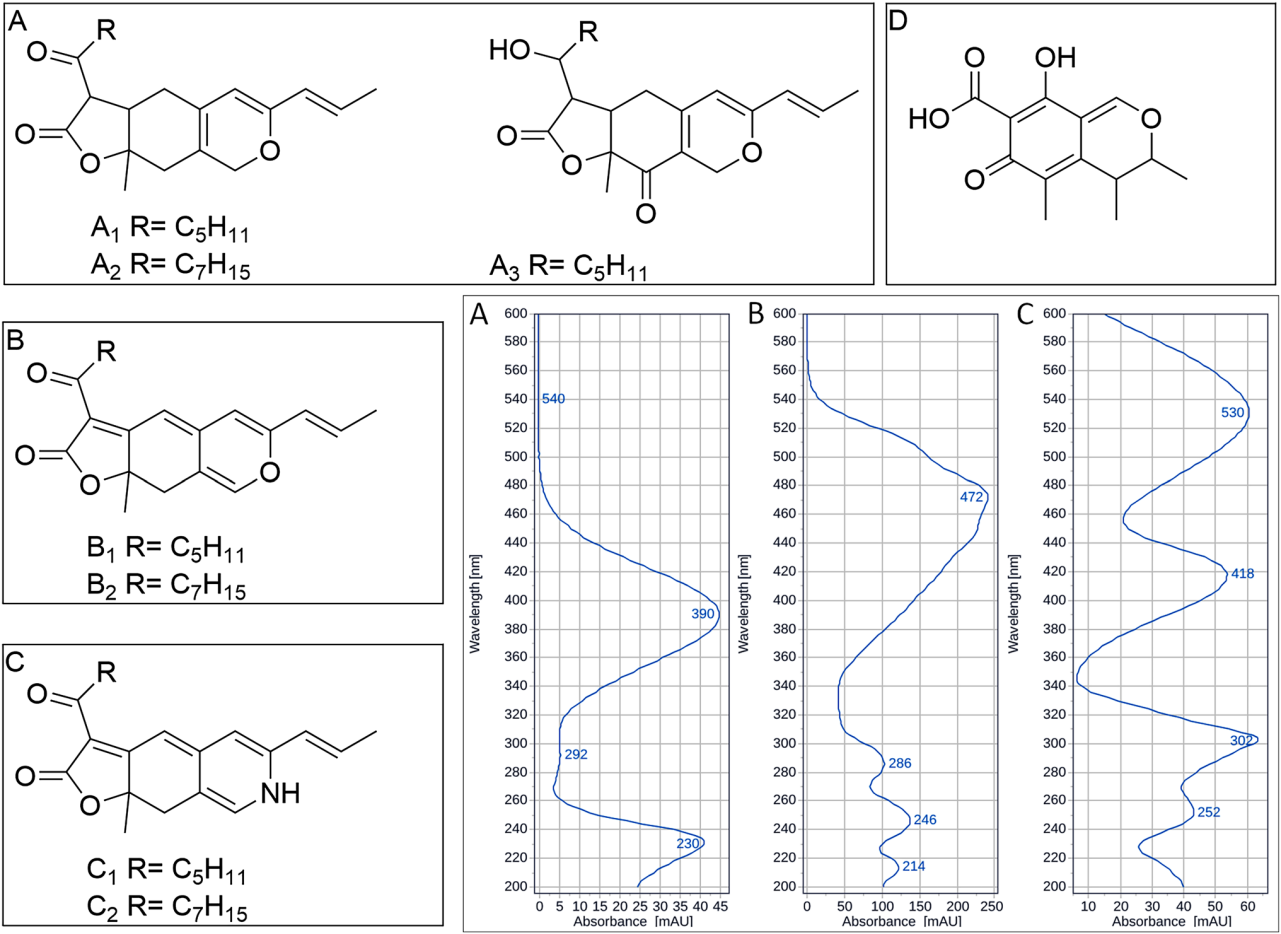
Chemical structures and absorption spectra of MPs and chemical structure of citrinin. **A** yellow MPs: **A**_**1**_ Monascin; **A**_**2**_ Ankaflavin; **A**_**3**_ Monascuspiloin; **B** orange MPs: **B**_**1**_ Rubropunctatin; **B**_**2**_ Monascorubrin; **C** Red MPS: **C**_**1**_ Rubropunctamine; **C**_**2**_ Monascorubramine; **D** Citrinin

MPs, oligoketides synthesized by the concerted action of polyketide synthase, fatty acid synthase and tailoring enzymes [6, 7], can be divided into three groups according to their colour: yellow with absorption maxima in the light region at 330–450 nm, orange with absorption maxima in the visible light region at 460–480 nm and red with absorption maxima in the visible light region at 490–530 nm [6]. Figure 1 shows six major MPs, i.e. monascin and ankaflavin (yellow), rubropunctatin and monascorubrin (orange), and rubropunctamine and monascorubramine (red) [8]. While yellow and orange MPs are biosynthesized by the fungus, red MPs result from the chemical reaction of orange MPs with compounds containing primary amino groups such as amino acids and peptides under neutral or mildly acidic pH [9]. This reaction, occurring during the cultivation or extraction, which is only conditioned by the availability of compounds with primary amino group in the substrate (e.g. rice) and suitable pH (within the range 5.0–8.0), may result in the formation of a mixture of red MP complexes derived from both basic red MPs, monascorubramine and rubropunctamine. This makes separation and identification of individual MPs from complex extracts very difficult. In addition, depending on the *Monascus* strain used and its biosynthetic capabilities together with culture conditions allowing various chemical reactions [9], MPs other than the basic ones shown in Fig. 1 can be formed by the *Monascus* species (more than 100 MPs have been described to date). Pigment profiles produced by different *Monascus* species may differ; one of the *Monascus* strains used in this study, *Monascus* sp. DBM 4361, produced monascuspiloin as its main MP, see Fig. 1.

The antimicrobial activity of MPs against various microorganisms has been reported in many studies [10–13] but their comparison is difficult because their authors worked with different mixtures of MPs in water, ethanol or other solvents. To summarize the current knowledge in this field, it seems that yellow MPs exhibit selective antimicrobial activity against Gram-positive bacteria [10, 14], orange MPs display antimicrobial activity against both Gram-positive and Gram-negative bacteria, such as *Staphylococcus aureus* [11, 12], *Escherichia coli* [15] and *Bacillus subtilis* [10] and red MPs and their amino acid derivatives are effective against bacteria, as well as against some filamentous fungi [11, 16–18]. It seems that the main effect of MPs can be expected on the surface of microbial cells, where they interact with the cell envelopes [12], disrupt membrane potential [19], prevent transport phenomena and oxygen access to the cells [16].

Main goal of the manuscript is to test whether MPs can be used as photosensitizers (PSs) in the antimicrobial photodynamic therapy (aPDT), which to the best of our knowledge, has never been explored. PSs are synthetic or natural compounds that can be activated by visible light at an appropriate wavelength, and, in the presence of oxygen, induce the release of reactive oxygen species (ROSs) that cause oxidative stress, leading to the death of eukaryotic or prokaryotic cells [20–22]. Currently, the aPDT is mainly studied using in vitro or animal models; in 2022, there were only 200 clinical trials reported [23]. The aPDT is suitable mainly for topical application to eradicate resistant microorganisms which can be the case of oral infections associated with the development of periodontal disease [24] or the treatment of non-healing skin wounds[25], including diabetic foot ulcers [26]. Since ROSs generated at aPDT could damage not only pathogenic microorganisms but also tissue host cells, studies have been conducted to address the cytotoxicity of aPDTs. In this sense, varying results can be found, depending on the PS used, its concentration, the design of the experiment and the cell line used [27–30]. For specific applications of aPDT, it is therefore necessary to find a combination of factors (PS concentration, light source, irradiation time) that lead to the desired effect on pathogens and at the same time have a minimal damaging effect on the treated tissue. In comparison with use of standard antibiotics, it may also exhibit time and dose-dependent cytotoxicity [31, 32]. Recently, photoantimicrobials have been proposed to control the spread of microbial pathogens on inanimate surfaces of nosocomial environments [33] and in feed and food packaging [34]. Indeed, PSs represent a class of compounds potentially relevant for sanitization in different fields: from clinical to industrial, from feed/food to agricultural ones.

Regarding MPs, till now, only the effectiveness of the orange MP rubropunctatin, as PS, was tested against cancer cells, where it showed a dual effect, both as standard anticancer drug (at dark) and as PS after light irradiation [35]. MPs are also known as antioxidants [36, 37]. Although it may seem odd to test antioxidants as PSs, it has already been shown [38] that the presence of antioxidants in photodynamic reaction can amplify cell damage. Actually, one single molecule can be both anti- and pro-oxidant dependending on circumstances of its application, a wonderful example might be curcumin [39], known as antioxidant and in the same time PS.

## Material and Methods

### Microbial Strains and Culture Conditions

*Monascus purpureus* DBM 4360 and *Monascus* sp. DBM 4361, originally isolated from non-sterile dried RYR samples used as the so-called colouring and functional hong-qu, respectively, were maintained on Sabourad agar (VWR Chemicals) slants at 4 °C. The strains are deposited in the Department of Biochemistry and Microbiology (DBM), University of Chemistry and Technology Prague. Both *Monascus* strains were cultured in SSC and SLC and the fermented substrate (rice in SSC) or mycelium (in SLC) were used to prepare *Monascus* extracts (MEs).

Rice was used as a typical SSC substrate for *Monascus* fermentation to produce RYR*.* Jasmine rice (Menu Gold) was washed with cold tap water, boiled in water for 15 min, drained and after 24 h, sterilized by autoclaving in autoclavable plastic bags. Sterile rice was inoculated by spore suspension (1 % v/w). Spore suspension was prepared covering the grown agar plates by physiological solution (9 g/L NaCl) and the agar surface was washed and scraped by the sterile inoculation loop to release the spores, spores were transferred to sterile tube and the suspension was used for inoculation. Rice was fermented for 8 and 14 days, respectively.

Different carbon and nitrogen sources were used in SLC performed in shaken 250 mL Erlenmeyer flasks (medium volume 100 mL), according to different requirements and behaviour of both strains (Table 1). The pH of culture media in SLC was adjusted before sterilization by autoclaving. Sterile media were inoculated by spore suspension (1 % v/v) and cultivated for 14 days at 30 °C on a rotary shaker (100 rpm).

**Table 1:** Cultivation conditions of *Monascus* strains

Strain	Sample name	Substrate/Medium composition (g/L)	Initial pH	Cultivation time (days)
*Monascus purpureus* DBM 4360	RR1	Jasmine rice (Menu Gold)	7	8
	RR2	Jasmine rice (Menu Gold)	7	14
	RM1	Glucose (Penta) 50, (NH_4_)_2_SO_4_ (Penta) 5, KCl (Penta) 0.5, KH_2_PO_4_ (Lach-Ner) 4, ZnSO_4_.7H_2_O (Penta) 0.01, MgSO_4_.7H_2_O (Roth) 0.5, FeSO_4_.7H_2_O (Lach-Ner) 0.01	5.5	14
	RM2	Maltose (Fluka) 20, casamino acids (Gibco) 5, K_2_HPO_4_ (Penta) 5, KH_2_PO_4_ (Lach-Ner) 5, CaCl_2_ (Lach-Ner) 0.1, MgSO_4_.7H_2_O (Roth) 0.5, trace elements solution 1 ml (FeSO_4_.7H_2_O (Lach-Ner) 10, ZnSO_4_.7H_2_O (Penta) 10, MnSO_4_.H_2_O (Lach-Ner) 3)	6.5	14
*Monascus* sp. DBM 4361	CR1	Jasmine rice (Menu Gold)	7	8
	CR2	Jasmine rice (Menu Gold)	7	14
	CM1	Rice starch (Sigma-Aldrich) 50, NaNO_3_ (Penta) 6.44, KCl (Penta) 0.5, KH_2_PO_4_ (Lach-Ner) 4, ZnSO_4_.7H_2_O (Penta) 0.01, MgSO_4_.7H_2_O (Roth) 0.5, FeSO_4_.7H_2_O (Lach-Ner) 0.01	5.5	14
	CM2	Maltose (Fluka) 20, casamino acids (Gibco) 5, K_2_HPO_4_ (Penta) 5, KH_2_PO_4_ (Lach-Ner) 5, CaCl_2_ (Lach-Ner) 0.1, MgSO_4_.7H_2_O (Roth) 0.5, trace elements solution 1 ml (FeSO_4_.7H_2_O (Lach-Ner) 10, ZnSO_4_.7H_2_O (Penta) 10, MnSO_4_.H_2_O (Lach-Ner) 3)	6.5	14

For the antimicrobial study, *Escherichia coli* MG 1655 [40], *Pseudomonas aeruginosa* PAO1 [41], *Bacillus subtilis* ATCC 6633 and *Staphylococcus aureus* ATCC 25923 were used. Bacteria were cultivated overnight at 37 °C in LB medium (g/L): tryptone (Sigma-Aldrich) 10, yeast extract (Merck) 5, NaCl (Penta) 5, agar (Carl Roth) 15 in the case of agar plates.

### MEs Preparation

RYR fermented by *Monascus* strains was extracted in acidified 85% ethanol (pH 4) in a rotary shaker for 40 min at 30 °C. The extraction ratio was 0.1 g of RYR/1 mL of extraction solution.

Grown mycelia were separated by filtration and extracted into acidified 85% ethanol (pH 4) on a rotary shaker for 40 min at 30 °C. The extraction ratio was 0.1 g of wet mycelium/1 mL of extraction solution.

Ethanol MEs (MPs and citrinin content is shown in Table S1) were evaporated on a rotary evaporator under reduced pressure at 40 °C (IKA RV 10 auto) and dissolved in DMSO (dimethylsulfoxide). During this step, the reaction between orange MPs and primary amino group-containing compounds (probably amino acids and peptides extracted together with MPs) resulted in the conversion of most orange MPs to their red analogues.

The designation of the MEs was chosen so that the first letter indicates the strain used, i.e. R for *M.* *purpureus* DBM 4360 and C for *Monascus* sp. DBM 4364, the second letter indicates the type of culture from which the MEs was obtained (R—SSC on rice, M—SLC, where the mycelium was extracted) and numbers were used for distinction of different culture conditions given in Table 1. (For example, ME designated as RR1 means that the MEs was obtained after the culture of *M. purpureus* DBM 4360 (R) on rice (R) and the culture lasted 8 days (1)).

### MEs Analysis

#### UHPLC Analysis

The MPs and citrinin content in MEs were analysed by UHPLC (Agilent Technologies 1260 Infinity II). The following conditions were used: Arion® Polar C18, 2.2 µm**,** 150 × 4.6 mm column; the mobile phase: 0.025% H_3_PO_4_ in water:acetonitrile in a ratio of 30:70; isocratic elution at a flow rate of 1 mL/min; injection volume 5 µL. For the determination of yellow, orange and red MPs, a photodiode detector set at 390, 470 and 500 nm resp. was used. For the determination of the mycotoxin citrinin, the fluorescence detector setting was 331 nm for excitation and 500 nm for emission.

Standards of the yellow MP monascin (Sigma‐Aldrich), orange MP rubropunctatin (1717 CheMall Corporation) and mycotoxin citrinin (Sigma‐Aldrich) were used as reference samples. A laboratory standard for rubropunctamine was prepared from the rubropunctatin standard by reaction with NH_4_OH (Penta) in 80 % ethanol. Unknown yellow, orange and red MPs, as well as monascuspiloin, were identified on the basis of their absorption spectra and quantified as equivalents to their respective standards, i.e. monascin, rubropunctatin and rubropunctamine.

#### Spectrophotometric Analysis and Construction of Irradiation Device

Absorption spectra of MEs were measured spectrophotometrically (Tecan reader Infinite pro) at 300–600 nm. In the case of an absorbance greater than 1, samples were diluted with DMSO and DMSO was used as a blank. The samples were measured before and after irradiation with blue light (100 mW/cm^2^, 100 J/cm^2^, 17 min, 6 cm distance).

The lighting unit (LULab) was designed by the University of Padua (Italy) and is equipped with a head composed by 25 high-power LEDs with a maximum emission peak at 410 nm. The system is powered by a specific PC-based control system, which allows the setting of irradiation time and irradiance values for a precise setting of the radiation fluence rate.

### Antimicrobial Assays

To evaluate the potential intrinsic dark toxicity of MEs, the following protocol was used. Overnight bacterial cultures were diluted with LB medium to obtain a concentration corresponding to 10^5^ CFU/mL, evaluated by a viable plate count method on LB agar plates. Each ME was serially two-fold diluted in order to reach the final concentrations summarized in Table S2. A volume of 100 µL of bacterial samples (10^5^ CFU/mL), 12 µL of diluted ME and LB medium were mixed in a 96-well plate to reach the final volume of 200 µL in each well. Cells in the mixture were incubated in 96-well plates at 37 °C for 24 h in the dark. After 24 h of incubation, cells were inoculated onto LB agar plates and incubated at 37 °C for 24 h in the dark. After incubation, the growth spots were evaluated and compared with untreated cells and cells treated with 6% DMSO (control for exclusion of solvent toxicity).

To evaluate the potential photoantimicrobial activity of MEs, cells were prepared and treated with serially diluted MEs as described above. After irradiation under light at 410 nm (100 mW/cm^2^, 100 J/cm^2^, 17 min, 6 cm distance), cells were incubated in 96-well plates at 37 °C for 24 h in the dark. After 24 h of incubation, cells were inoculated onto LB agar plates and incubated at 37 °C for 24 h in the dark. After incubation, growth spots were evaluated and compared with untreated cells and cells treated with 6% DMSO. For each experiment, the lowest concentration of serially diluted MEs that prevented the spot growth was considered as the MBC (minimal bactericidal concentration). All antimicrobial assays were performed in three biological replicates. The scheme of antimicrobial assay is visualized in Fig. S1A.

To evaluate the effects of possible irradiation products, 200 µL of each ME was transferred into 96-well plate and irradiated by blue light at 410 nm (100 mW/cm^2^, 100 J/cm^2^, 17 min, 6 cm distance). Then, irradiated MEs were administered to the cells and the potential intrinsic toxicity was evaluated as described above.

Additionally, four MEs (RR2, RM1, CR2 and CM1), each from different types of cultivation, were administered to microorganisms from the ESKAPE pathogens group, specifically *P. aeruginosa* and *S. aureus*. To evaluate the potential intrinsic toxicity of chosen MEs, the previously described protocol with particular changes, caused by higher sensitivity to DMSO and blue light, was used: 6 µL of MEs (only one—the highest, concentration); irradiation by blue light at 410 nm (100 mW/cm^2^, 30 J/cm^2^, 5 min, 6 cm distance); 10-fold dilution of grown cells (24 h of growth in 96-well plate); the number of living cells were evaluated by the viable count method on LB agar plates (24 h of growth). The scheme of antimicrobial assay is visualized in Fig. S1B.

### Statistical Analysis

To investigate the statistical significance (*P*-value < 0.05) of differences observed among MBC values of MEs in dark and under irradiating conditions, one-way ANOVA assay was performed using Excel software (MS Office) embedded module.

## Results

### Composition of *MEs*

**Fig. 2:**
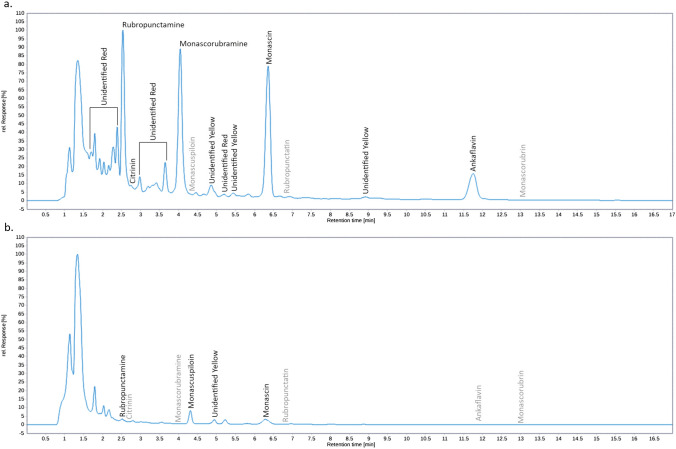
Typical ME chromatograms: **a** ME RM1 from *Monascus purpureus DBM 4360* mycelium; **b** ME CR2 from *Monascus* sp. DBM 4361 RYR

The MPs content in the MEs from mycelia or fermented rice determined by UHPLC analysis are shown in Table 2. As the used *Monascus* strains were originally isolated from different types of RYR (hong-qu), they not only produced different secondary metabolites’ profiles but also different quantities of MPs. *Monascus purpureus* DBM 4360, originated from the so-called colouring hong-qu, produced 6 main MPs shown in Fig. 1 and the total MPs content was 25 times higher and also the ratio of red to yellow MPs was higher compared to the *Monascus* sp. DBM 4361. Unfortunately, together with MPs, the *M. purpureus* DBM 4360 also produced citrinin unlike the other strain. *Monascus* sp. DBM 4361 produced monascuspiloin as its main yellow MP but its special feature, described previously in detail [13], was the ability to produce mainly MPs with a five-carbon side chain, formed by the incorporation of *β*-ketooctanoic acid into the basic structure of the MPs, i.e. monascin, rubropunctatin (see Fig. 1). *Monascus* sp. DBM 4361 can also produce monacolin K but not under the conditions used in this study. In chromatograms of MEs from both strains (Fig. 2), peaks belonging to MPs shown in Fig. 1 as well as to unidentified yellow and red MPs (recognized by typical spectra) were found.

**Table 2:** MPs and citrinin concentrations in extracts used for testing

Extracts	*Monascus purpureus* DBM 4360				*Monascus* sp. DBM 4361			
	RYR		Mycelium		RYR		Mycelium	
	RR1	RR2	RM1	RM2	CR1	CR2	CM1	CM2
Yellow MPs [mg/L]	1767.04	8591.65	1515.66	1582.70	59.82	533.71	1096.66	85.09
Orange MPs [mg/L]	–	–	–	–	–	–	8.53	–
Red MPs [mg/L]	4407.37	9646.76	21,545.80	867.16	18.50	73.10	273.50	–
Citrinin [mg/L]	3.88	5.32	1.65	4.37	–	–	–	–
Total MP content [mg/L]	6174.41	18,238.41	23,063.11	2449.86	78.31	606.81	1378.70	85.09
Red to yellow MPs ratio	2.49	1.12	14.22	0.55	0.31	0.14	0.25	

All MPs were quantified as monascin, rubropunctatin and rubropunctamine equivalents. (–) not detected. Cultivation and extraction were performed in triplicate (data not shown), one MEs parallel was chosen

### Antimicrobial Screening of MEs

To evaluate the potential bactericidal activity of the different MEs, two-fold decreasing serial dilutions of each ME were administered to the bacterial strains to find out the minimum bactericidal concentration, i.e. the concentration that completely prevented the growth of the tested microorganism on LB agar plate. A strategy to test crude extracts instead of pure compounds was used to screen potential candidates (MEs of different composition) for aPDT. This approach was chosen because the isolation of individual compounds from MEs is very difficult and will therefore only be carried out in the future for the MEs that showed the desired activity. Under the conditions tested, there was observed no bactericidal effect of DMSO, used as MEs solvent and the minimal inhibition concentration of DMSO was 12.5% for *E. coli* and *B. subtilis* and 6.25% for *P. aeruginosa* and *S. aureus*.

**Fig. 3:**
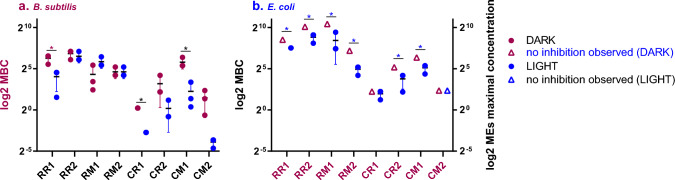
Antimicrobial activity of MEs and the maximal MEs concentrations used (different MEs from *M.* *purpureus* (R) and *Monascus* sp. (C); for their complete description see Table 2). Antimicrobial activity against *Bacillus subtilis* ATCC 6633 (**a**) and *Escherichia coli* MG1655 (**b**), under dark conditions and after irradiation is demonstrated as MBC values. All data are stated as the mean of at least three independent experiments and the bars represent standard deviations. The statistical analysis was performed using one-way ANOVA (* p < 0.05). Concentrations of MEs corresponding to the presented data are shown in Supplementary material—TableS2. The symbols represent: (•) the MBC values achieved under dark conditions; (•) the MBC values achieved under light conditions; (∆) the highest used concentration of MEs (6%) with no inhibition observed under dark conditions (values on the secondary y-axis), concentrations corresponding to 6% of MEs was used for one-way ANOVA statistical analysis; (∆) the highest used concentration of MEs (6%) with no inhibition observed under light conditions (values on the secondary y-axis), concentration corresponding to 6% of MEs was used for one-way ANOVA statistical analysis

Before testing MPs as PSs, the standard antimicrobial activity of the prepared MEs (i.e. without irradiation) was studied to find out their dark toxicity values. While the Gram-positive model bacterium, *B. subtilis*, showed a certain degree of sensitivity to the MEs even under dark conditions (Fig. 3A), no bactericidal effect was found in Gram-negative model bacterium *E. coli*, even at the highest MPs concentration in MEs (Fig. 3B) under dark conditions. In Fig. 3, the logarithmic yield (log2) of MBC concentrations, allowing easier comparison of the antimicrobial activity of individual MEs, was chosen because of the two-fold serial dilution of MEs used and also because of the order of magnitude different concentrations of MPs in the extracts obtained after cultivation of both *Monascus* strains.

**Fig. 4:**
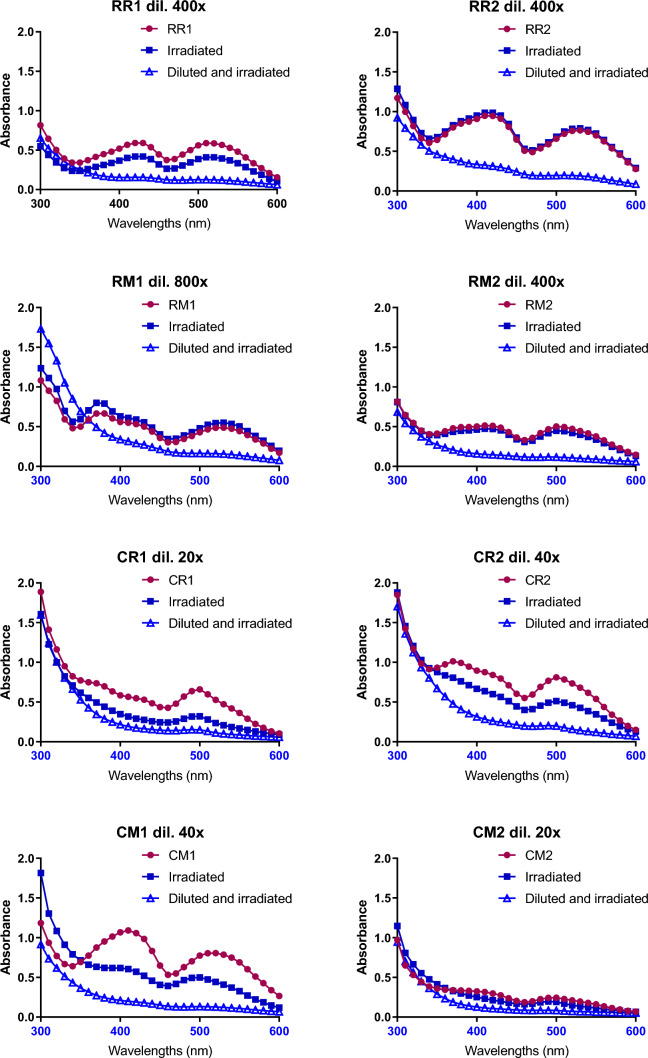
Absorption spectra of MEs before and after irradiation with blue light (410 nm, 100 mW/cm^2^, 100 J/cm^2^, 17 min, 6 cm distance). The difference between the effect of light on concentrated and diluted MEs is shown to demonstrate different behaviours of diluted and concentrated MEs upon irradiation. Before measurement, each ME was diluted with DMSO to reach an absorbance value under 1. The dilution ratio is stated on the figure for each ME. The curves represent absorbance spectra of (•) untreated MEs; (▪) irradiated MEs; (∆) firstly diluted and then irradiated MEs

Subsequently, to evaluate the potential photosensitizing activity of MEs, their absorption properties were investigated. In the absorption spectra of most MEs (Fig. 4), there is a peak in the blue region close to the ultraviolet band, which is the reason why a LED with maximum emission peak at 410 nm was chosen as the light source. Under the conditions tested, a bactericidal effect of mere blue light irradiation was not observed in any of the tested bacterial strain (data not shown). In *E.* *coli*, when the MEs were photoactivated under light at 410 nm (100 J/cm^2^), a bactericidal effect was observed with all the tested MEs except for CM2 (Fig. 3B). In the Gram-positive model *B. subtilis*, the blue light irradiation induced a further significant (*P* < 0.05) antimicrobial activity for RR1, CR1 and CM1 extracts compared to the corresponding dark toxicity values (Fig. 3A).

Encouraged by the results reached for model Gram-positive/Gram-negative organisms, effects of four MEs (RR2, RM1, CR2 and CM1) were tested on growth of *P. aeruginosa* and *S. aureus* (shown in Table S3). The intrinsic toxicity of DMSO and toxicity of blue light was excluded by carrying out control tests. In the case of *S. aureus*, the cells were very sensitive to the presence of MEs in the dark and the cells exhibited growth only in the presence of extract CM1. After irradiation of this CM1 extract, no growth was observed. On the other hand, the Gram-positive bacterium *P. aeruginosa* was tolerant to the presence of MEs under dark conditions and showed decrease in population density of 1–2 log CFU/ml compared to control. After irradiation, the population density decrease was in the range of 2–4 log CFU/mL compared to the dark conditions and again as well as in the previous tests, MEs from *Monascus* sp. DBM 4361 were more effective.

### MEs Photoactivity Assay

**Fig. 5:**
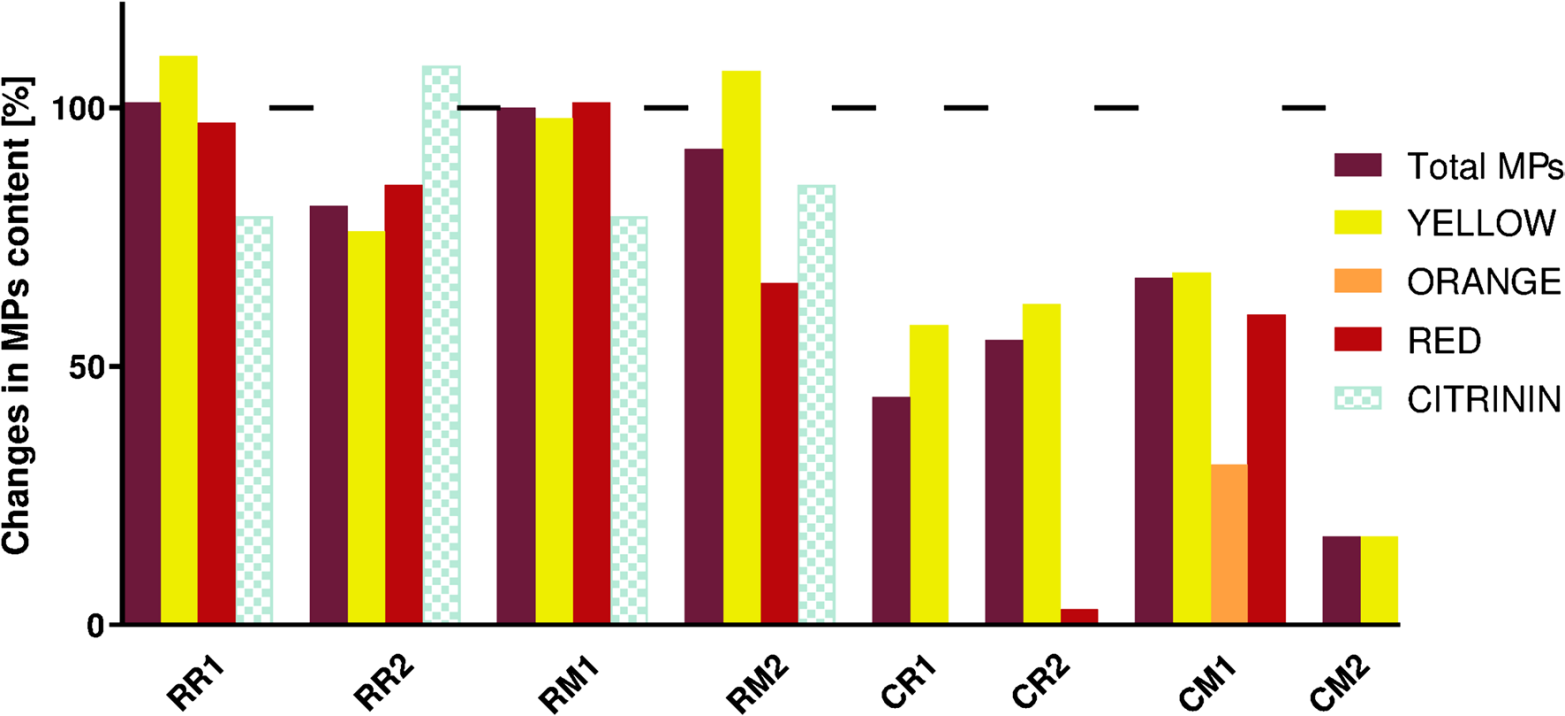
Changes of MPs and citrinin content after irradiation, as determined by UHPLC analysis, in percent. Values of 100% correspond to concentrations before irradiation, on the x-axis, there are different ME from *M. purpureus* (R) and *Monascus* sp. (C); for their complete description see Table 2

In the photodynamic field, the ideal PSs should display a low rate of photobleaching in order to maximize the antimicrobial effect. Thus, a spectrophotometric analysis was performed to determine if irradiation at 410 nm (100 J/cm^2^) could influence the absorbance of MEs as potential PSs. As can be observed in Fig. 4, the absorption spectra of undiluted MEs from *Monascus purpureus* DBM 4360 before and after irradiation were identical except for ME RR1, where a slight shift down is visible. On the other hand, all MEs from *Monascus* sp. DBM 4361 showed a decrease in the absorbance. The effects on diluted MEs were overall similar and the absorbance significantly decreased after irradiation when the typical peaks disappeared. Thus, the changes in MP content in samples after irradiation were determined by UHPLC analysis and the results are shown in Fig. 5. While the composition of *M. purpureus* MEs was only relatively little affected by irradiation, for *Monascus* sp. MEs the concentration of both yellow and red MPs dropped by about half. In general, a significant photochemical reaction was observed after irradiation of diluted samples (Fig. 4) and with *Monascus* sp. MEs (Fig. 5).

To exclude the toxicity of possible irradiation products, separately irradiated MEs were administered to the bacterial cells. These irradiated MEs had similar or lower antimicrobial activities against *E.* *coli* and *B. subtilis* (data not shown) in comparison with the effect of MEs and blue light acting directly in bacterial suspensions, which was standard experiment set-up.

## Discussion

After SSC and SLC of *Monascus purpureus* DBM 4360 and *Monascus* sp. DBM 4361, eight MEs were prepared, and their antimicrobial activities and photosensitizing potential were tested. The tested MEs differed in composition and total MPs concentration according to the previous expectation, based on experience with these strains [3, 13]. Within this work, all tested MEs exhibited a selective intrinsic, dark toxicity against *B. subtilis*, chosen as a Gram-positive model, which seems to be in agreement with the previously published results [10–12, 14, 16–18]. Although antimicrobial activity of MPs or their mixtures was found also for Gram-negative bacteria in the previous studies [15–18], in this study only weak or no activity was observed.

Information is limited regarding the mode of MP antimicrobial activity [11, 16]. Nevertheless, the observed selectivity could be ascribable to the different organization of cell wall of Gram-positive and Gram-negative bacteria. The higher susceptibility of Gram-positive bacteria observed in our study indicates the possible binding of MPs to available peptidoglycan structures of Gram-positive bacteria, while the effect of the outer membrane of Gram-negative bacteria is sufficient to protect them from the negative effect of MPs [42]. This is in agreement with the hydrophilic nature of red MPs abundant in the samples tested. Similarly, yellow monascuspiloin, prevailing in MEs from *Monascus* sp. DBM 4361, which is the most hydrophilic among yellow MPs detected (owing to the extra ketone group, see Fig. 1), may be efficiently excluded from the Gram-negative cell having a hydrophobic outer layer.

Moreover, the antimicrobial effect of MEs from *Monascus purpureus* DBM 4360 could be partially related to the presence of mycotoxin citrinin, which exhibits some antibiotic properties [43]. Although, the concentration of citrinin in MEs from *Monascus purpureus* DBM 4360 was very low (1.65–5.32 µg/mL in original MEs), while the MIC against *E. coli* and *B. subtilis* were found to be 50 and 100 µg/mL, respectively [44], its potential synergistic effect with MPs must be taken into account. Since no trace of mycotoxin citrinin has been detected in MEs from *Monascus* sp. DBM 4361, their observed antimicrobial activity in *B. subtilis* should be caused by other components. The biological activity of the *Monascus* sp. DBM 4361 major yellow MP—monascuspiloin has only partly been investigated in biological activity studies not related to its antimicrobial activity (just the study targeted to improvement of alcoholic liver injury of mice [45] and the study focussed on combination ionizing radiation and monascuspiloin administration in human prostate cancer therapy [46, 47]).

Dark cytotoxicity of MPs towards mammalian cell lines were studied in the couple of studies differing in experiment arrangements. Yellow (ankaflavin, monascin) and orange (rubropunctatin, monascorubrin) MPs did not exhibit cytotoxicity towards rat hepatocytes in vitro [10]; rubropunctatin was less cytotoxic compared to taxol for gastric epithelial cells [48]; red and yellow MPs were not cytotoxic for human keratinocytes and erythrocytes [49]; monaphilones A–C produced by *M. purpureus* NTU 568 were not cytotoxic to human lung cell lines [50]. Unfortunately, the cytotoxicity of MPs after irradiation has never been studied and will have to be completed if particular application of aPDT in human medicine is planned.

A novelty of this study is to investigate the potential photosensitizing power of MEs. The known natural PSs vary in their chemical structure but usually are heterocyclic compounds with many conjugated double bonds and several of them have been isolated from plants, fungi or bacteria [21]. MPs as polyketide compounds with 2 or 3 conjugated double bonds remind the natural PSs. MEs tested in this study were better photosensitizing agents for *E. coli* than for *B. subtilis*. In *E. coli*, the antimicrobial activity of MEs was observed only upon irradiation with blue light. A similar result, albeit with one concentration tested, was obtained for ESKAPE G^+^/G^−^ pathogens, *S. aureus* and *P. aeruginosa*, which is very valuable given the increasing resistance to traditional antibiotics in these pathogens. Thus, without displaying any intrinsic dark toxicity, MEs played the role of an ideal PS for Gram-negative bacteria, especially in case of MEs obtained from *Monascus* sp. DBM 4361. In this case, the hypothesized photooxidative stress occurring at cell wall level could increase the ability of MPs to overcome the hydrophobic barrier of outer membranes and induce the bactericidal effects. It is a question for future research which compound(s) in the *Monascus* sp. DBM 4361 is/are responsible for the PS effect, whether it might be a single compound, e.g. monascuspiloin, unique combination of MPs in these MEs or whether there is another compound acting as antioxidant, such as dimerumic acid produced by some *Monascus* strains [51], a siderophore, triornicin-like compound [52], which could enhance the photodynamic effect. This has to be clarified in future research.

Considerable photochemical reaction was observed with irradiation of diluted samples and in MEs with a lower MP content. This is in agreement with the literature, where the so-called dilution effect of MPs was described, it means that the molar absorption coefficient varies as a function of dilution factor in water environment [53]. It has been shown that yellow MPs are more stable than red ones [54, 55], and that red MPs in water solution are degraded by light. The stability of red MPs is affected by the ability to form electron-donor complexes. However, electron-donor complexes might be dissociated by water and are generally very unstable in polar solvents [53]. This might explain decreased stability of yellow MPs in MEs of *Monascus* sp. DBM 4361, formed mainly by hydrophilic monascuspiloin. In addition, red MPs possess higher antioxidant activity compared to yellow MPs [49], and may therefore protect other compounds in the solution. This might explain why more concentrated MEs from *M.* *purpureus* containing higher concentrations of red MPs are less efficient after irradiation compared to *Monascus* sp. ones. The (partial) MP degradation after irradiation also agrees with the study of Zheng et al. [35], where the degradation of rubropunctatin after irradiation by visible light (500 W halogen lamp, 4h) was detected.

It was hypothesized that MPs accumulate on the surface of bacterial cells and interfere with the membrane transport [11, 16] and recently it was demonstrated that in fungi they disrupt membrane potential [19]. Thus, it seems that MPs interact mainly with cell surfaces what seems to be a benefit for the potential photosensitizing activity of MEs. The light can affect MEs localized outside of the cells and their light-induced degradation may cause higher cell surface damage compared to dark treatment. However, the photostability of individual MPs and complex MEs, as well as their degradation after irradiation, requires further research.

## Conclusion

To the best of our knowledge, this is the first study to evaluate the potential of MPs in aPDT. Our screening experiments confirmed *Monascus* spp., isolated from the functional hong-qu (RYR), as a promising source of antimicrobials and demonstrated their potential as photoantimicrobials for aPDT. After irradiation by blue light, substantially increased antimicrobial activity of *Monascus* spp. MEs against Gram-negative bacteria was observed which seems to be great promise in possible aPDT due to high efficiency at low PS doses. Our findings open up the possibility of MPs use in aPDT, however, further investigation of the effect of light on MPs and the photoactivation of individual MPs is needed as well as implementation of cytotoxicity studies for particular applications.

### Supplementary Information

Below is the link to the electronic supplementary material.

Supplementary file1 (DOCX 9447 KB)

## Data Availability

The data underlying this article can be made available on reasonable request to the corresponding author.

## Code Availability

No codes have been created as part of this research.
