# Supplementary material for: Screening Antibacterial Photodynamic Effect of Monascus Red Yeast Rice (Hong-Qu) and Mycelium Extracts
Source: Curr Microbiol. 2024 May 21;81(7):183. doi: 10.1007/s00284-024-03725-6 (PMC11108928; doi:10.1007/s00284-024-03725-6)

**Screening antibacterial photodynamic effect of *Monascus* red yeast rice (hong-qu) and mycelium extracts**

**Current Microbiology**

**Marketa Husakova^1^, Viviana Teresa Orlandi^2^, Fabrizio Bolognese^2^, Barbora Branska^1^, Petra Patakova^1*^**

^1^ Department of Biotechnology, University of Chemistry and Technology Prague, Technicka 5, 160 00 Prague, Czech Republic

^2^ Department of Biotechnologies and Life Sciences, University of Insubria, Via JH Dunant 3, 21100 Varese, Italy

^*^Corresponding author: Department of Biotechnology, University of Chemistry and Technology Prague, Technicka 5, 160 00 Prague, Czech Republic. petra.patakova@vscht.cz

**Supplementary materials**

**Table S1.** MPs and citrinin concentrations in original ethanol extracts. All MPs were quantified as monascin, rubropunctatin, and rubropunctamine equivalents. (-) not detected. Cultivation and extraction was performed in triplicate (data not shown), one MEs parallel was chosen.

|  | ***Monascus purpureus* DBM 4360** | | | | | | ***Monascus* sp. DBM 4361** | | | | |
| --- | --- | --- | --- | --- | --- | --- | --- | --- | --- | --- | --- |
|  | **RYR** | | | **mycelium** | | | **RYR** | | | **mycelium** | |
|  | **RR1** | **RR2** | **RM1** | | **RM2** | **CR1** | | **CR2** | **CM1** | | **CM2** |
| **Yellow MPs [mg/L]** | 240.27 | 786.16 | 518.88 | | 181.20 | 7.01 | | 48.94 | 31.57 | | 17.91 |
| **Orange MPs**  **[mg/L]** | 56.40 | 682.73 | 1308.08 | | 8.36 | - | | - | 7.75 | | - |
| **Red MPs [mg/L]** | 206.86 | 289.72 | 31.41 | | 36.66 | 9.34 | | 12.98 | - | | - |
| **Citrinin [mg/L]** | 3.27 | 8.30 | 1.37 | | 3.00 | - | | - | - | | - |
| **Total MP content [mg/L]** | 503.53 | 1758.61 | 1858.37 | | 226.22 | 16.35 | | 61.92 | 39.32 | | 17.91 |

**Table S2**. Total pigment and citrinin content. Calculations of working concentrations prepared by two-fold serial dilution.

|  |  | **[mg/L]** | | | | | | | |
| --- | --- | --- | --- | --- | --- | --- | --- | --- | --- |
| **Binary serial dilution** | **%** | **RR1** | **RR2** | **RM1** | **RM2** | **CR1** | **CR2** | **CM1** | **CM2** |
|  | 100 | 6174.41 | 18238.41 | 23063.11 | 2449.86 | 78.31 | 606.81 | 1378.7 | 85.09 |
| **1** | 6 | 370.46 | 1094.30 | 1383.69 | 146.99 | 4.70 | 36.41 | 82.72 | 5.11 |
| **2** | 3 | 185.23 | 547.15 | 691.84 | 73.50 | 2.35 | 18.20 | 41.36 | 2.55 |
| **3** | 1.5 | 92.62 | 273.58 | 345.92 | 36.75 | 1.17 | 9.10 | 20.68 | 1.28 |
| **4** | 0.75 | 46.31 | 136.79 | 172.96 | 18.37 | 0.59 | 4.55 | 10.34 | 0.64 |
| **5** | 0.375 | 23.15 | 68.39 | 86.48 | 9.19 | 0.29 | 2.28 | 5.17 | 0.32 |
| **6** | 0.1875 | 11.58 | 34.20 | 43.24 | 4.59 | 0.15 | 1.14 | 2.59 | 0.16 |
| **7** | 0.0938 | 5.79 | 17.10 | 21.62 | 2.30 | 0.07 | 0.57 | 1.29 | 0.08 |
| **8** | 0.0469 | 2.89 | 8.55 | 10.81 | 1.15 | 0.04 | 0.28 | 0.65 | 0.04 |

**Table S3.** Antimicrobial activity of chosen MEs on *Pseudomonas* *aeruginosa* PAO1 and *Staphylococcus* *aureus* ATCC 25923 evaluated as viable count. Assays were performed in three biological replicates.

| **[CFU/mL]** | | ***Pseudomonas aeruginosa PAO1*** | | ***Staphylococcus aureus ATCC 25923*** | |
| --- | --- | --- | --- | --- | --- |
| **EXTRACTS** | | **DARK** | **LIGHT** | **DARK** | **LIGHT** |
| ***Monascus purpureus* DBM 4360** | **RR2** | 10^10^ | 10^9^ | no growth | no growth |
|  | **RM1** | 10^10^ | 10^9^ | no growth | no growth |
| ***Monascus* sp. DBM 4361** | **CR2** | 10^9^ | 10^7^ | no growth | no growth |
|  | **CM1** | 10^9^ | 10^8^ | 10^8^ | no growth |
| **Control** | | 10^11^ | 10^11^ | 10^9^ | 10^9^ |

**Fig. S1.** The experimental set-up scheme of the antimicrobial assays; A: Set-up for antimicrobial activity tests against *B. subtilis* and *E. coli* B: Set-up for antimicrobial activity tests against *P. aeruginosa* (*PA*) and *S. aureus* (*SA*).
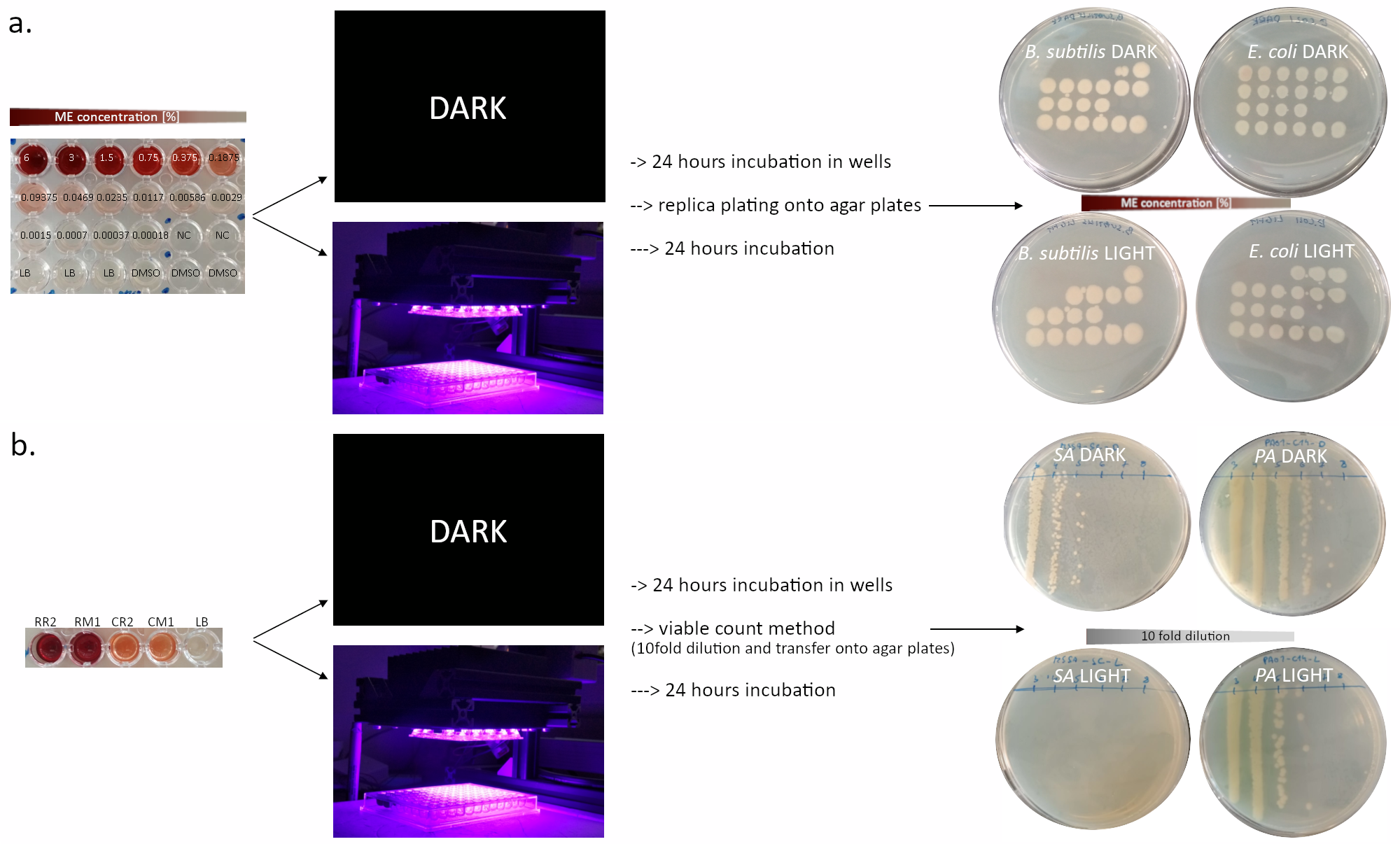

Supplement: Supplementary file 1 — Supplementary file1 (DOCX 9447 KB) [file 284_2024_3725_MOESM1_ESM.docx]
